# Morphological, Transcriptomic and Hormonal Characterization of Trimonoecious and Subandroecious Pumpkin (*Cucurbita maxima*) Suggests Important Roles of Ethylene in Sex Expression

**DOI:** 10.3390/ijms20133185

**Published:** 2019-06-28

**Authors:** Yunli Wang, Chundong Yan, Bingxue Zou, Chaojie Wang, Wenlong Xu, Chongshi Cui, Shuping Qu

**Affiliations:** 1 Key Laboratory of Biology and Genetic Improvement of Horticultural Crops (Northeast Region), Ministry of Agriculture/Northeast Agricultural University, Harbin 150030, China; 2 College of Horticulture and Landscape Architecture, Northeast Agricultural University, Harbin 150030, China

**Keywords:** *Cucurbita maxima*, transcriptome sequencing, floral sex expression, ethylene signal synthesis and transduction, chemical treatment

## Abstract

Sex expression is a complex process, and in-depth knowledge of its mechanism in pumpkin is important. In this study, young shoot apices at the one-true-leaf stage and 10-leaf stage in *Cucurbita maxima* trimonoecious line ‘2013–12’ and subandroecious line ‘9–6’ were collected as materials, and transcriptome sequencing was performed using an Illumina HiSeq^TM^ 2000 System. 496 up-regulated genes and 375 down-regulated genes were identified between shoot apices containing mostly male flower buds and only female flower buds. Based on gene ontology (GO) and Kyoto Encyclopedia of Genes and Genomes (KEGG) analysis, the differentially expressed genes were mainly enriched in the ethylene and auxin synthesis and signal transduction pathways. In addition, shoot apices at the 4-leaf stage were treated with the ethylene-releasing agent 2-chloroethylphosphonic acid (Ethrel), aminoethoxyvinyl glycine (AVG), AgNO_3_ and indoleacetic acid (IAA). The number of female flowers up to node 20 on the main stem of ‘2013–12’ increased significantly after Ethrel and IAA treatment and decreased significantly after AVG and AgNO_3_ treatment. The female flowers in ‘9–6’ showed slight changes after treatment with the exogenous chemicals. The expression of key genes in ethylene synthesis and signal transduction (*CmaACS7*, *CmaACO1*, *CmaETR1* and *CmaEIN3*) was determined using quantitative RT-PCR, and the expression of these four genes was positively correlated with the number of female flowers in ‘2013–12’. The variations in gene expression, especially that of *CmaACS7*, after chemical treatment were small in ‘9–6’. From stage 1 (S1) to stage 7 (S7) of flower development, the expression of *CmaACS7* in the stamen was much lower than that in the ovary, stigma and style. These transcriptome data and chemical treatment results indicated that IAA might affect pumpkin sex expression by inducing *CmaACS7* expression and indirectly affecting ethylene production, and the ethylene synthesis and signal transduction pathways play crucial roles in pumpkin flower sex expression. A possible reason for the differences in sex expression between pumpkin lines ‘2013–12’ and ‘9–6’ was proposed based on the key gene expression. Overall, these transcriptome data and chemical treatment results suggest important roles for ethylene in pumpkin sex expression.

## 1. Introduction

In flowering plants, sex expression is an important process that differentiates male and female flowers. Many factors are involved in the complex process of sex expression, such as temperature, daylength, ethylene, auxin, cytokinin, brassinolide (BR) and nitrogen metabolism [1]. In addition to environmental factors, key genes also play a crucial role in sex determination. In the Cucurbitaceae, such as melon (*Cucumis melo* L.) and cucumber (*Cucumis sativus* L.), floral primordia are initially bisexual, and sex determination occurs by developmental arrest of either the stamen or the carpel whorl, resulting in unisexual flowers [2]. Genes specifically expressed in the stamen or carpel primordia control the development of male, female, and hermaphrodite flowers [3,4,5,6].

Because the Cucurbitaceae includes seven sex forms, it is a model dicotyledonous plant family for sex determination research. The sex determination mechanism of Cucurbitaceae has been thoroughly studied, and ethylene is a key hormone that promotes female flower development in Cucurbitaceae plants. The enzymes 1-aminocyclopropane-1-carboxylate (ACC) synthetase (ACS), which catalyzes the rate-limiting step in ethylene biosynthesis, and ACC oxidase (ACO), which converts ACC into ethylene, are key in ethylene biosynthesis [7]. The genes *CsACS2* (the *M* (*andromonoecious*) loci in cucumber), *CmACS7* (the *M* loci in melon), *ClACS7* and *CitACS4* can inhibit stamen development in female flowers and determine andromonoecy [3,4,8,9,10,11,12]. *CsACS11* (the *A* (*androecy*) loci in cucumber) and *CmACS11* loss-of-function mutants lead to male plants [5,10]. Cucumber plants harboring *CsACS1G* (the *F* (female) loci) bear only female flowers, although the molecular mechanisms remain to be investigated [6,13]. *CitACS4 is* expressed specifically in carpel primordia in watermelon (*Citrullus lanatus* sp.), which indicates that *CitACS4* also plays a role in sex determination [12]. *CsACO2* is essential for carpel development and interacts with *CsACS11* to promote female flower development in cucumber [14].

Ethylene signaling is perceived by a family of ethylene receptors. *CS-ETR2* and *CS-ERS*, which are ethylene receptor-related genes, may play roles in the development of female flowers in gynoecious cucumbers under high levels of endogenous ethylene [15]. *ETR1* is known as the negative ethylene perception gene. *CsETR1* was demonstrated to be involved in stamen development in female cucumber flowers through the induction of DNA damage [16]; *etr1-1* transgenic melon plants had a higher number and earlier appearance of carpel-bearing flowers on the main stem. This phenomenon indicates that *ETR1* also plays a role in sex determination in melon. Recently, with the help of yeast one-hybrid and ChIP-PCR assays, *CsERF110* (ethylene-responsive factor 110) and *CmERF110* were shown to respond to ethylene signaling and enhance *CsACS11* and *CmACS11* promoter activity in cucumber and melon [17]. *CsERF31* (ethylene-responsive factor 31) also responded to the ethylene signal derived from *F* and positively regulated ethylene feedback by activating *M* expression in cucumber [18].

Pumpkin, a monoecious plant that belongs to the genus *Cucurbita*, is an annual vine. Pumpkin includes the three most economically important cultivated species, pumpkin (*Cucurbita maxima* Duch.), zucchini (*Cucurbita pepo* L.) and squash (*Cucurbita moschata* Duch.). Hybrids show strong heterosis, but hybrid production requires cross-pollination every year. Therefore, it is necessary to manually remove males, which takes time and effort. Thus, it is important to explore the sex determination mechanism of pumpkin to provide a basis for pumpkin cultivation techniques. Typical monoecious pumpkin varieties have three different developmental phases: only male flowers are produced in the first phase, the production of female and male flowers alternates during the second phase, and only female flowers are produced in the third phase [19]. According to genetic and physiological analyses of sex expression, the androecious phenotype of *C. pepo* is determined by a single recessive gene [20], while gynoecious form is a dominant characteristic [21]. Androecious *C. pepo* is ethylene insensitive, and this trait is controlled by an ethylene-response pathway gene named *weak ethylene insensitive* (*CpWEI*) [22,23]. Two constitutive triple response (CTR) genes, *CpCTR1* and *CpCTR2*, were cloned as negative regulators of ethylene signaling in flowers [24]. Through transcriptional regulation analysis, ethylene receptor and CTR-like genes were confirmed as negative regulators of female flower transition during the earlier stages of plant development [25]. In addition, *CpACS27A*, a gene homologous to *CmACS7* and *CsACS2*, was cloned and observed to regulate stamen development in *C. pepo* [26]. In recent years, a strong female developmental gene in *C. maxima* was mapped to an interval of 35.2 Kb, and the possible candidate gene was identified as a pentatricopeptide repeat-containing family gene [27].

RNA sequencing (RNA-seq) is a useful transcriptome profiling tool for rapid gene discovery, exploring gene expression, elucidating phylogenetic relationships, molecular marker development and related gene expression analyses that has been used in *Cucurbita* species [28,29,30]. In 2011, the first *Cucurbita* transcriptome was generated using 454 GS FLX Titanium technology [28]. Subsequently, many differentially expressed genes associated with flowering, disease resistance, fruit size, fruit shape, fruit color, fruit sweetness, fruit nutritional qualities, and chilling sensitivity were determined using RNA-seq [29,31,32,33,34,35,36].

The sex expression mechanism in pumpkin is less well known than that in other Cucurbitaceae species, such as cucumber and melon. Therefore, in this study, RNA-seq was carried out using subandroecious and trimonoecious *C. maxima* as materials, and differentially expressed genes involved in the ethylene synthesis and signal transduction pathways were identified. The number of female flowers in trimonoecious *C. maxima* changed significantly after 4 chemical treatments, and the expression levels of *CmaACS7*, *CmaACO1*, *CmaETR1* and *CmaEIN3* were positively correlated with the number of female flowers. The number of female flowers in subandroecious *C. maxima* changed slightly, and the variations in gene expression, especially that of *CmaACS7*, were small in amplitude. The expression of *CmaACS7* in the ovary, stigma and style at different stages of flower development indicated that *CmaACS7* plays an important role in sex differentiation. The transcriptome data and results of chemical treatment indicated that the ethylene synthesis and signal transduction pathways play a crucial role in pumpkin flower sex expression.

## 2. Results

### 2.1. Flower Phenotype

**Figure 1:**
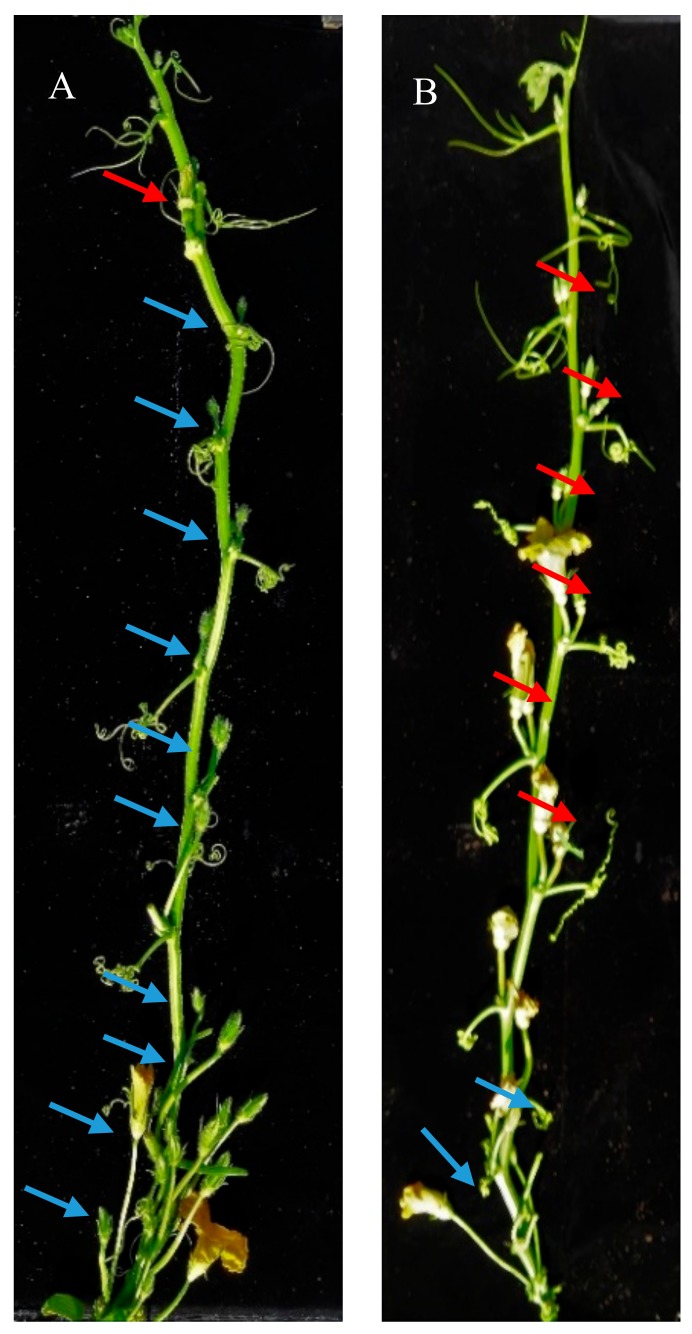
Different flower phenotype of two materials. (**A**) Flower phenotype of ‘9–6’. (**B**) Flower phenotype of ‘2013–12’. ‘9–6’ and ‘2013–12’ produced female flowers 4% and 51% respectively when observed up to node 20 on the main stem. Blue arrows indicated male flowers and red arrows indicated female flowers.

Flowers on the main stem of the subandroecious line ‘9–6’ were mainly male unisexual flowers; however, one or two female flowers appeared at nodes 15–16; fewer than 10% female flowers were observed up to node 20 of the main stem. Several male and bisexual flowers were present at the base of the main stem in the trimonoecious line ‘2013–12’, and all flowers produced after the first female flower, which appeared at node 10, were female unisexual flowers. The female flower proportion of ‘2013–12’ was approximately 50% up to node 20 on the main stem (Figure 1). Considering pumpkin hybrid production, the seed production process could be simplified by using the ‘2013–12’ inbred line.

### 2.2. RNA-Seq of Pumpkin

In ‘9–6’, shoots at one-true-leaf stage (LS1) (MS1-type sample) contained only male flower buds, whereas those at 10-leaf stage (LS10) (MS10-type sample) contained male flower buds and one or two female flower buds. In ‘2013–12’, shoots at LS1 (FS1-type sample) contained male flower buds and one or two bisexual flower buds, and those at LS10 (FS10-type sample) contained only female flower buds. There were obvious sex expression differences in FS1 vs. FS10 and in MS10 vs. FS10, but no significant differences were observed in MS1 vs. MS10 and MS1 vs. FS1.

In this research, the molecular mechanism of sex expression was explored using an RNA-seq approach; young shoots of ‘2013–12’ and ‘9–6’ at the LS1 stage and LS10 stage were used as materials. Through sequencing, a total of 525.41 Mb raw reads and 509.23 Mb clean reads, which showed valid bases from 96.22% to 97.17%, were obtained. In the clean reads of all 12 samples, the Q30 scores ranged from 93.89% to 94.60%, and the GC content ranged from 44.5% to 45% (Table 1), which demonstrated that high-quality sequencing results were obtained for further research.

All clean reads were subsequently subjected to de novo assembly with the Trinity program and produced 747,168 unigenes with a total length of 969.15 Mb. The average number of unigenes was 1297.09 bp. Of these, the longest unigene was 20,293 bp, and the shortest unigene was 201 bp (Table S1). Approximately 81.34–82.78% of clean reads could be mapped to the reference genome (Table S2). A total of 131,960 unigenes (17.66%) were annotated based on the information available from five public protein databases, i.e., the NR, Swiss–Prot, KOG, GO and Kyoto Encyclopedia of Genes and Genomes (KEGG) databases, using BLAST with an E-value cut-off of 1.0e^−5^. In total, 57,118 unigenes, 43.28% of the total assembled unigenes, were matched in the NR database, and 38,487 (29.17%), 32,257 (24.44%), 13,828 (10.48%), and 33,927 (25.71%) of the unigenes were matched in the Swiss–Prot, KOG, GO and KEGG databases, respectively.

**Figure 2:**
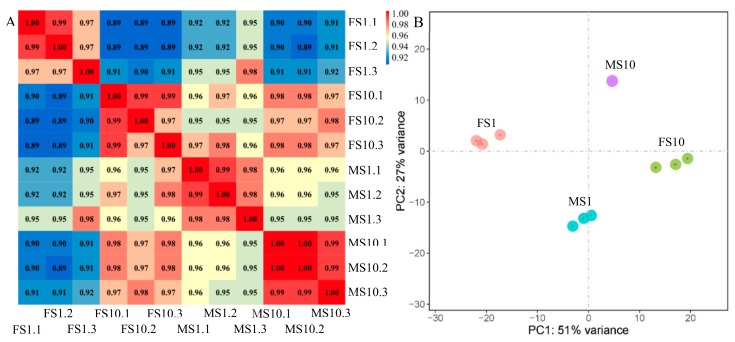
Correlation analysis and principal component analysis of samples. (**A**) Correlation analysis of gene expression between samples. Comparisons of gene expression between replicates on the x-axis and those on the y-axis. The correlation coefficients between replicate samples were approximately 1. The correlation coefficient was show as a color value (blue: less similar, and red: more similar). (**B**) Principal component analysis plot of RNA sequencing (RNA-seq) data in samples. Three biological replicates for each sample type were clustered together. Blue plots indicated samples of ‘9–6’ at one-true-leaf stage (LS1) (MS1), purple plots indicated samples of ‘9–6’ at 10-leaf stage (LS10) (MS10), pink plots indicated samples of ‘2013–12’ at LS1 (FS1), and green plots indicated samples of ‘2013–12’ at LS10 (FS10).

The correlation of gene expression between samples is important to test the reliability of the database and the rationality of sample selection. According to the unigene expression level, the correlation coefficients between replicate samples were approximately 1 (Figure 2A), and the PCA results showed that the three biological replicates of each sample type were clustered together (Figure 2B). Similar results were obtained through gene expression distance analysis (Figure S1). Overall, the similarity of expression profiles and the high correlation between replicates indicated high data reliability of the high-throughput sequencing and reproducible replicate samples in this study.

### 2.3. Differential Expression Analysis and Functional Annotation

**Figure 3:**
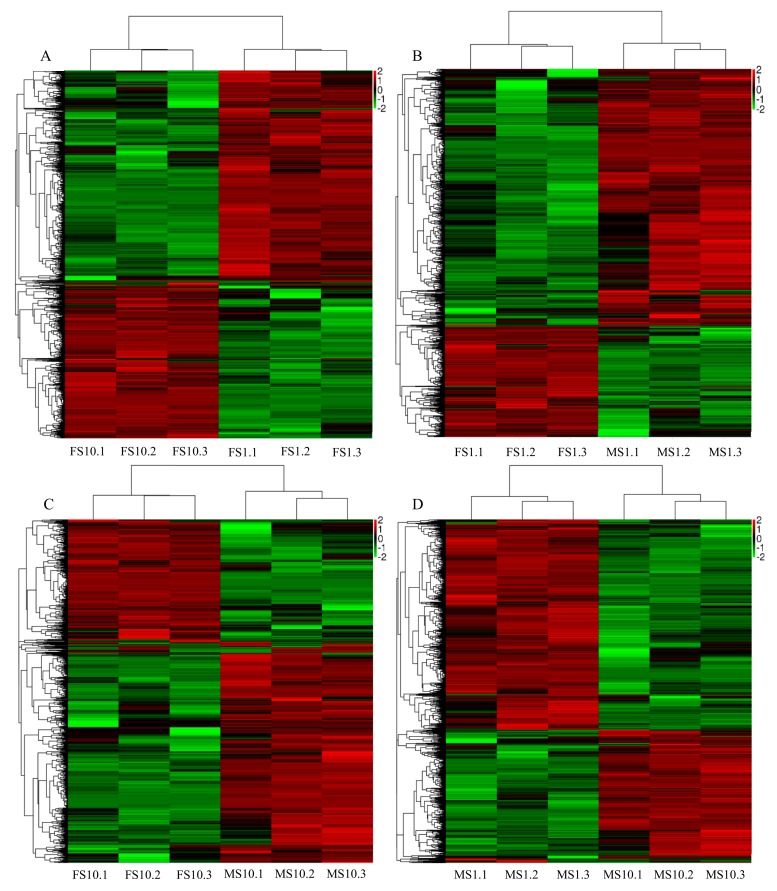
Heatmap visualization of expression profiles of MS1, MS10, FS1 and FS10. (**A**) Heatmap visualization of expression profiles between FS1 and FS10. (**B**) Heatmap visualization of expression profiles between FS1 and MS1. (**C**) Heatmap visualization of expression profiles between FS10 and MS10. (**D**) Heatmap visualization of expression profiles between MS1 and MS10. Red arrow, > two-fold up-regulation; green arrow, > two-fold down-regulation.

**Figure 4:**
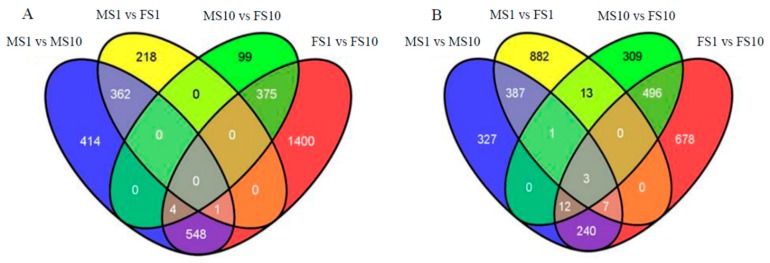
Number of differentially expressed genes among MS1, MS10, FS1 and FS10. (**A**) Number of down-regulated expressed genes. (**B**) Number of up-regulated expressed genes. MS1 indicated samples of ‘9–6’ at LS1, MS10 indicated samples of ‘9–6’ at LS10, FS1 indicated samples of ‘2013–12’ at LS1, and FS10 indicated samples of ‘2013–12’ at LS10.

To characterize transcriptional variations that occur in response to sex determination, the transcripts between two different sample types were compared, and the number of differentially expressed genes (DEGs) including > two-fold up-regulation or > two-fold down-regulation was counted. log_2_FoldChange and *p*-value of DEGs between two sample types (FS1 vs. MS1, FS10 vs FS1, MS10 vs. MS1, FS10 vs. MS10) were listed in Non-published Material 1. As shown in Figure 3, 2306 genes were differentially expressed in MS1 vs. MS10 (977 were up-regulated and 1329 were down-regulated), 3764 genes were differentially expressed in FS1 vs. FS10 (1436 up-regulated and 2328 down-regulated), 1874 genes were differentially expressed in MS1 vs. FS1 (1293 up-regulated and 581 down-regulated), and 1312 genes were differentially expressed in MS10 vs. FS10 (834 up-regulated and 478 down-regulated). A total of 4327 DEGs were sample-specific. Amounts of 414, 218, 99 and 1400 DEGs were specifically down-regulated expressed, while 327, 882, 309 and 678 DEGs were specifically up-regulated expressed in the MS1 vs. MS10, MS1 vs. FS1, MS 10 vs. FS1 and FS1 vs FS10 comparisons, respectively (Figure 4). In total, 871 DEGs (496 up-regulated and 375 down-regulated) were differentially expressed in both FS1 vs. FS10 and MS10 vs. FS10 and were not differentially expressed in MS1 vs. MS10 or MS1 vs. FS1. These genes showed tissue-specific expression between shoot apices containing male flower buds and female flower buds, which indicated that these genes may be involved in sex determination.

**Figure 5:**
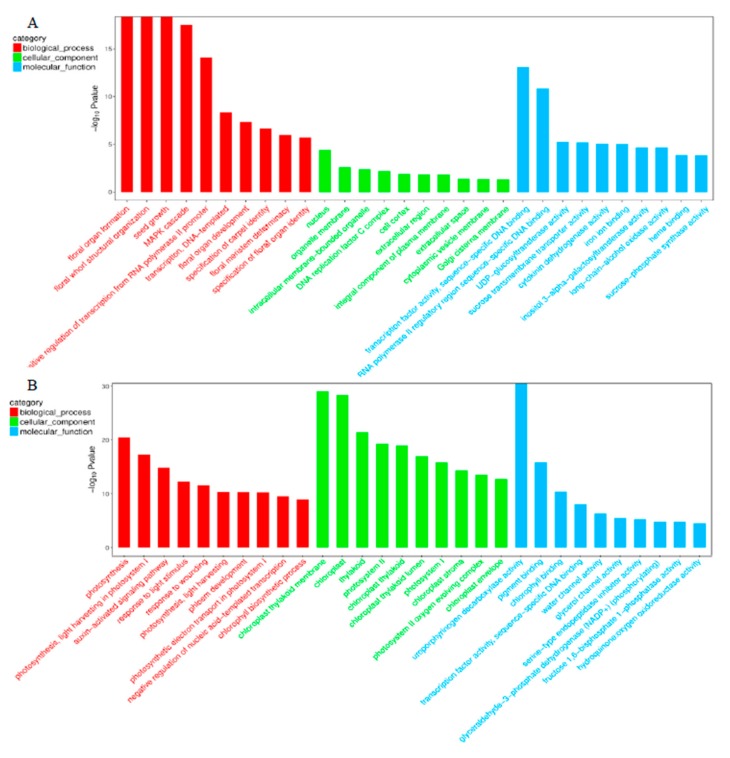
The gene ontology classification of up-regulated genes (**A**) and down-regulated genes (**B**) between young shoot apices containing male flower buds and female flower buds.

**Figure 6:**
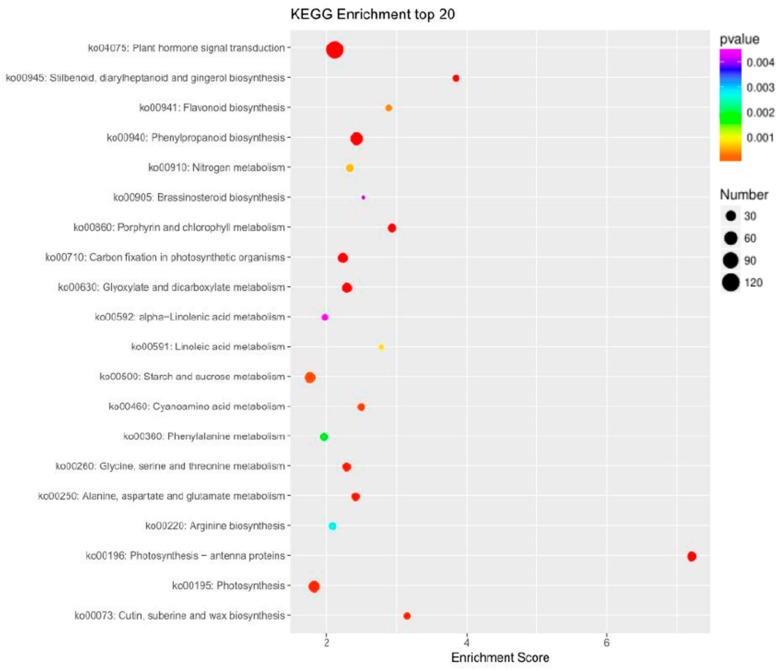
Kyoto Encyclopedia of Genes and Genomes (KEGG) pathway scatterplot of 871 differentially expressed genes (DGEs). The x-axes indicate enrichment score. The bigger the bubble, the more the DGEs. The smaller the p-value, the more significant the KEGG enrichment.

To further characterize the genes identified as being differentially expressed in association with sex, 871 DEGs were mapped to GO classifications, and KEGG enrichment was determined. The GO annotations of the DEGs are shown in Figure 5. The main functional groups of the up-regulated genes are related to floral organ formation, mitogen-activated protein kinase (MAPK) cascade, floral organ development, specification of carpel identity and floral meristem determinacy. The main functional groups of the down-regulated genes are related to photosynthesis regulation, the auxin-activated signaling pathway, wounding, phloem development and other processes. The 20 most highly represented pathways of the 871 DEGs are shown in Figure 6. The DEGs were mainly enriched in plant hormone signal transduction, stilbenoid, diarylheptanoid and gingerol biosynthesis, flavonoid biosynthesis, phenylpropanoid biosynthesis, nitrogen metabolism and other pathways. The majority of the DEGs were involved in plant hormone signal transduction pathway (largest point in Figure 6), and the expression of these DEGs was significantly expression between the shoot apices contained different number of female flower buds (red point, which indicated *p*-value < 0.001). The expressed of these DEGs was significantly different between the shoot apices containing different numbers of female flower buds (red point, which indicated *p*-value < 0.001). This result indicated that pumpkin floral organ formation in the main stem is controlled through changes in the transcript levels of key genes involved in hormone signal transduction.

**Figure 7:**
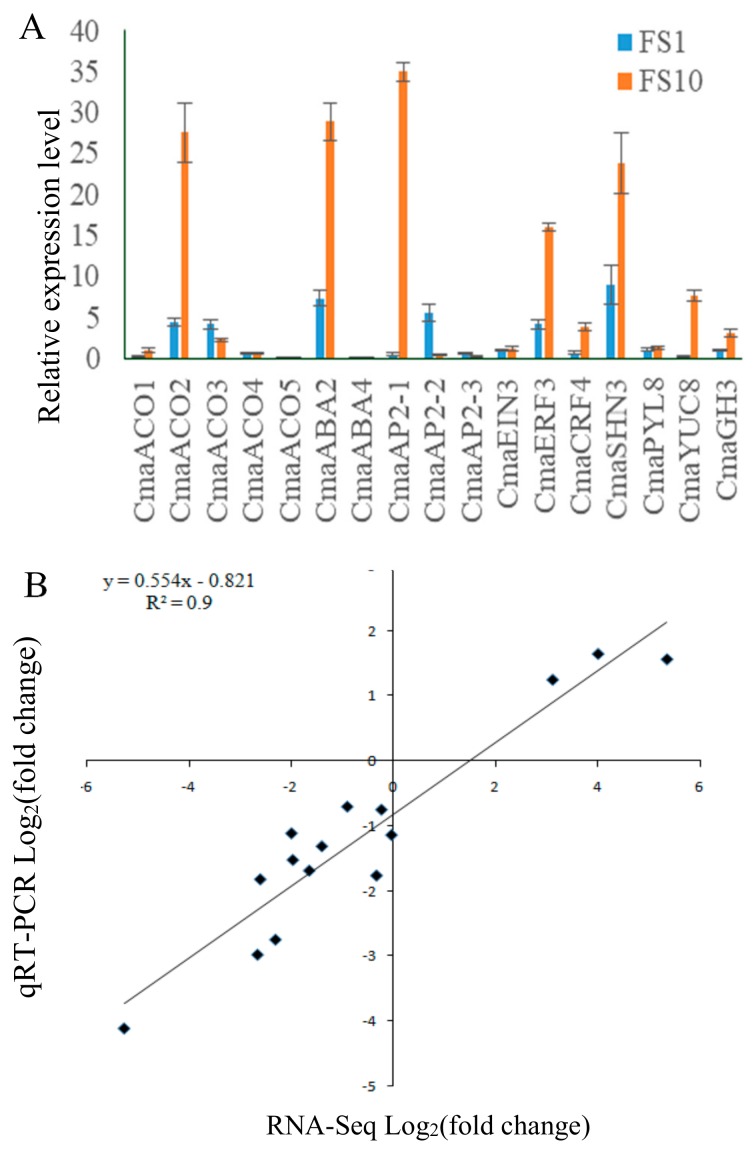
Expression comparison of 17 selected genes using RNA-seq and quantitative reverse transcription polymerase chain reaction (qRT-PCR). (**A**) Relative expression of 17 selected genes in FS1 and FS10 using qRT-PCR. The *CmaACO1* expression at FS10 was assumed as 1. Data are displayed as the ratio of expression to *CmaActin* with three biological replicates. Error bars represent standard error (SE). The qRT-PCR of primers used are given in Table S3. (**B**) Comparison of the expression ratios of 17 selected genes in ‘2013–12’ at LS1 and LS10 stage using RNA-seq and qRT-PCR.

To further test the reliability of RNA-seq, 17 genes were randomly selected from the 871 DEGs based on sequencing, and quantitative reverse transcription polymerase chain reaction (qRT-PCR) analysis was performed with specific primers using FS1 and FS10 samples. The results showed that the gene expression patterns of FS1 vs. FS10 examined by qRT-PCR analyses were similar to those revealed by RNA-seq (Figure 7A). Most of the expression patterns shown by qRT-PCR agreed with the RNA-seq ratios, with a relative coefficient of *R*^2^ = 0.9 (Figure 7B).

### 2.4. Hormone Genes Associated with Sex Expression

According to the KEGG pathway results, the majority of DEGs were enriched in the plant hormone signal transduction pathway related to ethylene synthesis and signal transduction. Based on log_2_-fold change >1 or <−1 in FS1 vs. FS10 and MS10 vs. FS10 as specific queries, 25 DEGs are listed in Table 2. Two genes are associated with ethylene synthesis, and 23 genes are associated with the ethylene signaling pathway. Among these 23 genes, six are also associated with auxin, abscisic acid, cytokinin, gibberellin, jasmonic acid and salicylic acid. Ethylene synthesis genes and most positively regulated ethylene signaling genes were up-regulated, and three negatively regulated ethylene signaling genes (*CmaCh01G009990*, *CmaCh01G016830* and *CmaCh09G005960*) were down-regulated in FS10. These results indicated that genes related to ethylene synthesis and signal transduction positively regulate female sex expression in pumpkin. Two ethylene synthesis genes, *CmaCh03G005620* and *CmaCh02G000660*, were analyzed for expression in different organs. *CmaCh02G000660* (named *CmaACO1*) showed flower-specific expression and significantly differential expression between female and male flowers (Non-published Material 2), so we selected it for further study.

### 2.5. Plant Phenotypes after Chemical Treatments

To further demonstrate the effects of ethylene and auxin on the sex expression of pumpkin, shoot apices were treated with four chemicals: Ethrel, aminoethoxyvinyl glycine (AVG), AgNO_3_ and indoleacetic acid (IAA). Table 3 shows the effects of these chemicals on sex expression in ‘9–6’ and ‘2013–12’. The subandroecious ‘9–6’ control produced female flowers on 4% of the 20 nodes, and the first female node was node 15.4. There was no obvious increase or decrease in the number of female flowers, and the appearance of female flowers was not significantly advanced or delayed after Ethrel, IAA, AVG or AgNO_3_ treatment compared with the control. The trimonoecious ‘2013–12’ control produced female flowers at 51% of the 20 nodes and bisexual flowers at 27.5% of the nodes, and the first female node was node 10.1. When Ethrel was applied to the shoot apices at the four-leaf stage, female flowers were produced at 72.5% of the nodes, and bisexual flowers were produced at 3% of the nodes. The first female node was node 5.9, implying a significant increase in female flower development and a significant decrease in bisexuality as well as a distinctly early appearance of female flowers relative to the control. IAA treatment exerted a similar effect to Ethrel. When AgNO_3_ was applied, female flowers were produced at 33.5% and bisexual flowers were produced at 33.5% of the nodes, and the first female node was node 13.7, implying a significant decrease in female flower development. AVG treatment had a similar effect to that of AgNO_3_.

### 2.6. Gene Expression under Chemical Treatment

**Figure 8:**
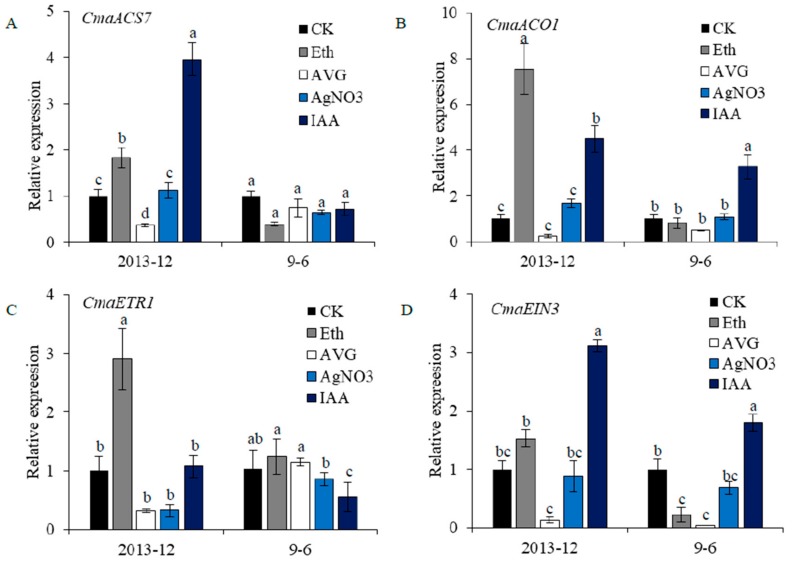
Expression changes of *CmaACS7* (**A**), *CmaACO1* (**B**), *CmaETR1* (**C**) and *CmaEIN3* (**D**) after Ethrel (Eth), aminoethoxyvinyl glycine (AVG), AgNO_3_ and indoleacetic acid (IAA) treatment. The expression level of *CmaACS7*, *CmaACO1*, *CmaETR1* and *CmaEIN3* were detected using qRT-PCR. Gene expression of control (CK) ‘2013–12’ was assumed as 1 in chemical treatment ‘2013–12’, and control (CK) ‘9–6’ was assumed as one in chemical treatment ‘9–6’. Data are displayed as the ratio of expression to *CmaActin* with three biological replicates. Error bars represent standard error (SE). The qRT-PCR of primers used are given in Table S3. Phytohormone levels followed by the same lowercase letter are not significantly different at *p* < 0.05 using SSR’s test.

The transcriptome data and exogenous chemical treatment results shown in our research indicated that the ethylene synthesis and signal transduction pathways play a crucial role in pumpkin flower sex expression. Thirteen *ACS* genes in pumpkin were cloned, and gene expression patterns in different tissues were analyzed. Among them, *CmaACS7* (*CmaCh10G007020*) was specifically expressed in female flower buds (Non-published Material 3). *CmaACO1* was significantly up-regulated in FS10 in the RNA-seq data, and its female-specific expression was confirmed using qRT-PCR. *CmaETR1* (*CmaCh05G007380.1*) and *CmaEIN3* (*CmaCh04G024920*) were highly homologous to *ETR1* and *EIN3* in melon and cucumber, and were significantly highly expressed in the shoot apices of ‘2013–12’ compared with ‘9–6’ [37]. To examine whether these four chemicals could regulate the expression of ethylene synthesis and signal transduction genes, the expression levels of *CmaACS7*, *CmaACO1*, *CmaETR1* and *CmaEIN3* were detected using qRT-PCR. As shown in Figure 8, the chemicals caused different degrees of change in the expression of the 4 genes. Compared to the ‘2013–12’ control, the expression level of *CmaACS7* was elevated significantly after Ethrel and IAA treatment, whereas the mRNA level was significantly reduced after AVG treatment. AgNO_3_, an inhibitor of the ethylene biological response, had a nonsignificant effect on *CmaACS7* expression. These results indicated that *CmaACS7* expression in ‘2013–12’ could be affected by chemical treatment. However, in the ‘9–6’ control, the mRNA levels of *CmaACS7* were not significantly changed after the 4 chemical treatments (Figure 8A). As shown in Figure 8B, compared to those in the ‘2013–12’ control, the mRNA levels of *CmaACO1* were significantly elevated after Ethrel and IAA treatment, whereas the mRNA level was reduced after AVG treatment. Compared to the ‘9–6’ control, the mRNA level of *CmaACO1* was significantly elevated after IAA treatment but slightly reduced after AVG treatment. As shown in Figure 8C, compared to the ‘2013–12’ control, the mRNA level of *CmaETR1* was significantly elevated after Ethrel treatment, whereas the mRNA levels were reduced after AVG and AgNO_3_ treatment. Except for the IAA treatments, the tested chemicals had no effect on *CmaETR1* expression compared to the ‘9–6’ control. As shown in Figure 8D, compared to the ‘2013–12’ control, the mRNA levels of *CmaEIN3* were significantly elevated after IAA treatment but significantly reduced after AVG treatment. Compared to the ‘9–6’ control, the mRNA level of *CmaEIN3* was significantly elevated after IAA treatment, whereas the mRNA levels were reduced after Ethrel and AVG treatment. In ‘2013–12’, most of the expression changes in *CmaACS7*, *CmaACO1, CmaETR1* and *CmaEIN3* were positively correlated with the percentage of female flowers, which indicated that exogenous chemicals can affect female flower number on the main stem through gene expression changes in the ethylene synthesis and signal transduction pathways. Compared with those in ‘2013–12’, the variations in gene expression in ‘9–6’, especially that of the upstream regulator *CmaACS7*, were small in amplitude.

### 2.7. CmaACS7 Expression at Different Flower Development Stages

**Figure 9:**
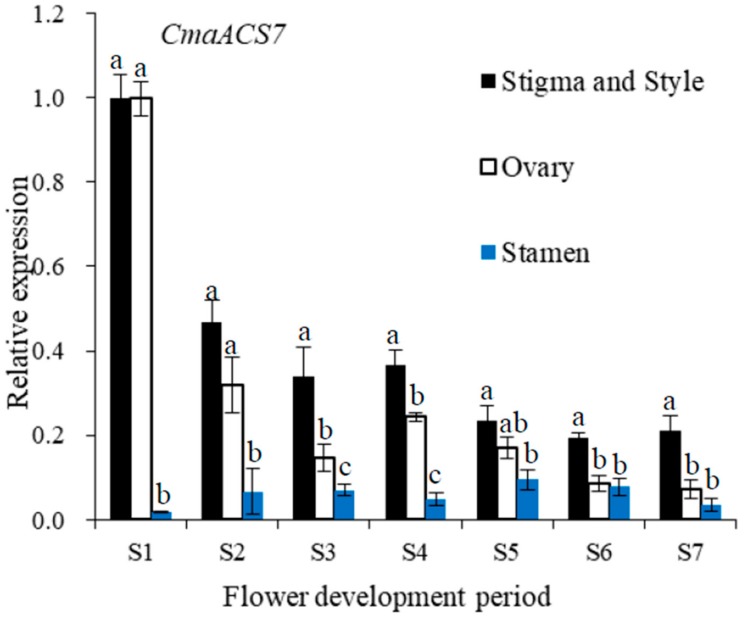
Relative expression of *CmaACS7* at different stages (S1-S7) of ‘2013–12’ flower development using qRT-PCR analyses. The *CmaACS7* expression at S1 ovary was assumed as unity. The relative expression level of *CmaACS7* in ovary, stigma and style was significantly high, while less expression was observed in stamen. Data are displayed as the ratio of expression to *CmaActin* with three biological replicates. Error bars represent standard error (SE). The qRT-PCR of primers used are given in Table S3. Phytohormone levels followed by the same lowercase letter are not significantly different at *p* < 0.05 using SSR’s test.

To examine whether *CmaACS7* could involve sex expression in pumpkin, the expression of *CmaACS7* in the stamen, ovary, stigma and style at different stages (S1–S7) was determined using qRT-PCR analyses. Pumpkin flower buds 7 days before flowering (dbf) (lengths of 2–4 mm) were considered to be at stage 1 (S1); 6 dbf (lengths of 6–8 mm), stage 2 (S2); 5 dbf (lengths of 10–12 mm), stage 3 (S3); 4 dbf (lengths of 15–17 mm), stage 4 (S4); 3 dbf (lengths of 20–25 mm and a yellow stigma), stage 5 (S5); 2 dbf (lengths of 30–35 mm), stage 6 (S6); and 1 dbf, stage 7 (S7). As shown in Figure 9, *CmaACS7* expression in the ovary, stigma and style decreased significantly during flower development, and the highest expression level was observed at S1. *CmaACS7* expression in the stamen increased over the first five developmental stages and then decreased; the highest expression level was observed at S5. *CmaACS7* expression in the stamen was much lower than that in the ovary, stigma and style throughout S1 to S7, and this lower expression of *CmaACS7* was significant at S1. The trend of *CmaACS7* expression shown in Figure 9 confirmed that *CmaACS7* has an important role in early sex differentiation.

## 3. Discussion

The experiments in this study were carried out with the subandroecious line ‘9–6’ and the trimonoecious line ‘2013–12’. Based on observations up to node 20 on the main stem, ‘9–6’ produced only one or two female flowers at the top node, while ‘2013–12’ produced approximately 10 consecutive female flowers. These two lines showed significant differences in flower sex and are therefore good materials for studying sex expression mechanisms.

The study of floral sex determination using RNA-seq has previously been reported in Cucurbitaceae plants, such as cucumber and melon [38,39,40], and crucial genes controlling floral sex have been cloned [3,4,6,9,10,11,13]. However, knowledge of the mechanisms and genes involved in sex expression in pumpkin is still urgently needed to provide additional information. In the present study, four sample types, i.e., shoot apices of ‘9–6’ and ‘2013–12’ at LS1 and LS10, were collected for RNA extraction to select DEGs that correspond to sex determination. Through transcript compilation, 871 DEGs potentially involved in pumpkin sex determination were selected. These DEGs were enriched in plant hormone signal transduction pathways, and most were related to ethylene and auxin synthesis and signal transduction. Our previous research results also showed that the endogenous ethylene and IAA contents in female flowers increased gradually to a level much higher than that in male flowers during pumpkin flower development [37]. These results confirmed that ethylene and auxin play crucial roles in pumpkin flower sex determination. According to transcriptome analyses, the major DEGs positively related to ethylene signaling exhibited high expression in the FS10 compared to the MS10 and FS1 transcriptomes (Table 2). These results indicated that ethylene signaling was more active in ‘2013–12’ than in ‘9–6’, and there was a positive correlation between ethylene-activated signaling and female flower proportion. Several DEGs related to ethylene signaling were also associated with auxin, abscisic acid, cytokinin, gibberellin, jasmonic acid and salicylic acid, demonstrating the intricate relationships among the signaling pathways of ethylene and other hormones. Other hormonal factors might also influence the transmission of ethylene signals related to sex expression.

Exogenous chemical treatment could induce plant sexual transition. In our research, the loss of bisexual flowers in ‘2013–12’ was accompanied by an increase in female flowers after treatment with the exogenous auxin IAA and the ethylene-releasing agent Ethrel. Some female flowers transformed from bisexual flowers retained underdeveloped stamens (Figure S2), distinct from normal female flowers. These results indicated that Ethrel and IAA treatment inhibited stamen development and led to sexual transition at the early flower development stage. Similar results were found for gynoecious and monoecious cucumber and melon [15,41,42]. AgNO_3_, an inhibitor of the ethylene biological response, and AVG, an inhibitor of ethylene biosynthesis, reduced the incidence of female flowers. These results are consistent with those for melon and cucumber [43,44] but in contrast to the results for watermelon [26,45]. Thus, the mechanisms of sex expression in pumpkin might be similar to those in melon and cucumber.

After the 4 different chemical treatments, a wide variation (14–21.5%) in female sex expression was observed in the trimonoecious line ‘2013–12’. In the subandroecious line ‘9–6’, the number of female flowers was not significantly changed after Ethrel, IAA, AVG or AgNO_3_ treatment. We noticed that the changes in flower sex in ‘2013–12’ were greater than those in ’9-6’ when the lines were treated with the same chemicals at the same concentration. According to our previous research, ’9–6’ shows delayed abscission of ethylene-treated male flowers and a weak triple response in five-day-old seedlings compared to those of ‘2013–12’ [27], indicating that both floral buds and seedlings had reduced sensitivity to ethylene. Therefore, we preliminarily speculated that ethylene signaling could be transduced in ‘9–6’ plants but was much weaker than that in ‘2013–12’. This result is probably because an important gene involved in ethylene accumulation or signal multiplication cannot act normally in ‘9–6’.

In ‘2013–12’, the expression of *CmaACS7*, *CmaACO1* and *CmaETR1* was significantly promoted by Ethrel. Ethrel could increase ethylene level and amplify ethylene signaling in the apex by upregulating the expression of ethylene synthesis and ethylene receptor genes, leading to a significant increase in female flowers (a 21.5% increase) and advancing the first female flower appearance. After IAA treatment, ‘2013–12’ showed a significant increase (16.5%) in female sex expression, a significant decrease (22%) in bisexuality, and advancement of the first female flower appearance; Ethrel treatment led to similar tendencies. The expression of ethylene biosynthetic genes (*CmaACS7*, *CmaACO1*) and ethylene signaling genes (*CmaEIN3*) was significantly increased. IAA treatment promoted ethylene production and the expression of ethylene-related genes. Similar conclusions have been reached in zucchini (*Cucurbita pepo* L.) and cucumber [46,47]. Auxins can induce ethylene production by stimulating the expression of *ACS*, causing fruit softening [48]. In our research, IAA treatment also induced a much higher level of *CmaACS7* expression. We suggest that IAA might affect pumpkin sex expression by inducing *CmaACS7* expression and indirectly affecting ethylene production. Ethylene- insensitive (EIN)3 is continuously degraded in plant cells [49], and the stability of EIN3 is regulated by the 26S proteasome [50,51]. IAA might affect the 26S proteasome to increase the stability of EIN3. Both AVG and AgNO_3_ treatment inhibited female sex expression and delayed the first female flower in ‘2013–12’, but they had different effects on gene expression. AVG treatment reduced the expression of the ethylene synthesis genes *CmaACS7* and *CmaACO1*, whereas AgNO_3_ reduced the expression of only the ethylene signal transduction gene. These results indicated that reduced ethylene levels and weakened ethylene signaling intensity could affect pumpkin sex expression.

Ethrel caused no significant changes in the expression of ethylene synthesis or receptor genes compared to the ‘9–6’ control, which indicated that ‘9–6’ plant had lacked its normal function of responding to Ethrel signal. It is interesting that, although the expression of *CmaACO1* and *CmaEIN3* changed under IAA treatment in ‘9–6’, the percentage of female flowers and the node of first female appearance were not significantly affected compared with those of the ‘9–6’ control. IAA had no influence on the expression of the upstream regulator *CmaACS7*. In fact, variations in *CmaACS7* expression were not obvious after Ethrel or other chemical treatments in ‘9–6’, which corresponded to no significantly changed number of female flowers. These results indicate that the expression level of *CmaACS7* plays a crucial role in sex expression in pumpkin. Activated ACS can be induced by ethylene and IAA to regulate downstream genes in cucumber [4]. In our research, *CmaACS7* in pumpkin ‘9–6’ lost its capability to respond to IAA or ethylene after chemical treatment. The sequences of CmaACS7 were highly similar to those of CsACS2 (87.47%) and CmACS7 (87.47%) [4,8]. Closely linked markers of *CmaACS7* were developed to analyze 200 F_2_ individuals derived from a cross of ‘2013–12’ and ‘9–6’. Notably, markers closely linked to *CmaACS7* were not genetically linked to female flower proportion. We speculated that another key gene influencing *CmaACS7* response might be mutated in ‘9–6’. 

Using F_2_ individuals derived from a cross between the trimonoecious line ‘2013–12’ and the subandroecious line ‘9–6’, a gene controlling high female flower proportion (higher than 45% up to node 20) was mapped and located in a narrow region. Several transcription factor genes were found in the located region, which did not contain any genes directly associated with ethylene synthesis and signaling transduction. Thus, we predicted that a transcription factor gene could be stimulated by hormone signaling (ethylene and IAA) and then the transcription factor protein could specifically be combined with *CmaACS7* promoter to led high expression of *CmaACS7* gene in ‘2013–12’ flowers. When the sequencing of transcription factor gene changed, *CmaACS7* gene could not be regulated. So *CmaACS7* had lost its positive feedback capability with respect to the ethylene pathway in ‘9–6’. This phenomenon could explain the different sex expression between pumpkin lines ‘2013–12’ and ‘9–6’, and ethylene sensitivity was reduced in ‘9–6’. Further research about the lack of increase female flowers after chemical treatment in ‘9–6’ is being planned to carry in our lab. The transcriptome data and chemical treatment results in our research benefited for key genes selection and gene interactions research regulated to pumpkin sex expression.

## 4. Materials and Methods

### 4.1. Plant Materials

The experiment was carried out with the subandroecious inbred line ‘9–6’ and the trimonoecious inbred line ‘2013–12’ (Figure 1). The seeds of ‘9–6’ and ‘2013–12’ were germinated at 30 °C in the dark for 36 h after being treated with 55°C water for 8 h and then transplanted to greenhouse breeding plots at Northeast Agricultural University. Four-leaf-stage seedlings were transferred to a greenhouse, and the required irrigation and fertilizer were applied under natural photoperiodic conditions in the spring of 2017.

### 4.2. RNA Extraction and RNA-Seq Processing

In subandroecious line ‘9–6’, shoots at LS1 contained only male flower buds, whereas those at LS10 contained male flower buds and one or two female flower buds. In trimonoecious line ‘2013–12’, shoots at LS1 contained male flower buds and one or two bisexual flower buds, and those at LS10 contained only female flower buds. There were obvious sex expression differences in FS1 vs. FS10 and in MS10 vs. FS10, which could explore the sex expression related genes. There was no significant differences were observed in MS1 vs. MS10 and MS1 vs. FS1, which could remove DEGs by virtue of different genetic background.

Young shoot apices of ‘9–6’ and ‘2013–12’ at LS1 and LS10 were collected for RNA extraction to study the transcriptome (four sample types with three biological replicates, 10 plants for each sample). All samples were immediately frozen in liquid N_2_ and were stored at −70 °C for RNA-seq analysis. Total RNA was were extracted using TRIzol reagent (Invitrogen, USA) and were treated with RNase-free DNase. mRNA was isolated from the total RNA using Dynabeads Oligo (dT) (Invitrogen Dynal), fragmented into small pieces and then copied into cDNA using reverse transcriptase. After purification, the cDNA fragments were connected using sequencing adapters. The fragments were sequenced from directions on an Illumina HiSeq^TM^ 2000 System (Illumina, San Diego, CA), and then the quality and purity of the constructed library were determined using an Agilent 2100 Bioanalyzer (Agilent Technologies, Palo Alto, CA, USA.) [52].

### 4.3. RNA-Seq Assembly, Annotation and Transcriptome Sequence Analysis

The raw reads from each library were assembled separately through base-calling analysis. After quality assessment using FASTQC software [53], the trimmed adapter sequences and the low-quality reads were removed using NGS QC TOOLKIT v2.3.3 [54]. The high-throughput clean reads were mapped to the Cucurbita maxima (Rimu) reference genome (ftp://www.cucurbitgenomics.org/pub/cucurbit/genome/Cucurbita_maxima/v1.1/Cmaxima_v1.1.chr.fa.gz) by the TopHat2 program [55]. The default settings were used for the remaining parameters. The uniformity and the saturation of sequencing data were analyzed based on the alignment results. Functional annotation of unigenes was performed by searching against the nonredundant (NR) (ftp://ftp.ncbi.nih.gov/blast/db), Swiss–Prot (http://www.uniprot.org/downloads) and clusters of orthologous groups for eukaryotic complete genomes (KOG) (ftp://ftp.ncbi.nih.gov/pub/COG/KOG/kyva) databases using BLAST with an E-value of 1e^−5^.

Unigene expression was determined based on fragments per kilobase per million reads (FPKM) [56]. To discover the relationships among the samples, principal component analysis (PCA) was performed using unigene expression. To study the similarity between samples, sample-to-sample distances were calculated using the Euclidean distance method. The samples belonging to the same experimental conditions were close in distance and grouped together preferentially.

Differentially expressed genes (DEGs) were identified with an adjusted *p*-value < 0.05 for multiple tests using the Benjamini-Hochberg method [57] for controlling the false discovery rate. The DEGs were functionally annotated by mapping onto GO classifications (using the adjusted *p*-value; http://www.geneontology.org/) using Blast2GO [58]. Three categories of GO annotations, i.e., biological process, molecular function, and cellular component, were analyzed. After the hypergeometric test, Bonferroni correction was employed for P-value correction with a cut-off of 0.05. KEGG pathways were identified and mapped according to *p*-values by searching against the KEGG database (http://www.genome.jp/kegg/pathway.html) [59].

### 4.4. Quantitative RT-PCR Analysis

Shoot apices of ‘2013–12’ at LS1 and LS10 were collected, and RNA was extracted for real-time quantitative polymerase chain reaction (qRT-PCR) analysis. cDNAs were reverse transcribed using 2 µg of total RNA with the PrimeScript RT Reagent Kit (Takara, Dalian, China). For qRT-PCR, 20 µL samples were run in triplicate on an ABI Prism 7000 Sequence Detection System and Applied Biosystems software using SYBR Green PCR Master Mix (Applied Biosystems, Carlsbad, CA, USA). Thermal cycling was performed using an initial denaturation step of 95 °C for 3 min followed by 40 cycles at 95 °C for 10 s, annealing temperature for 20 s, and 72 °C for 20 s. The relative expression levels of the genes were calculated by the 2^–ΔΔCt^ method and were normalized to the control gene actin. The primer sequences used in qRT-PCR are listed in Table S3.

### 4.5. Plant Hormone Treatments

To study the effects of ethylene and auxin on the sex of *C. maxima*, 4 different chemical solutions, i.e., 100 mg/L 2-chloroethylphosphonic acid (Ethrel) (an ethylene-releasing agent), 100 mg/L aminoethoxyvinyl glycine (AVG) (an inhibitor of ethylene biosynthesis), 200 mg/L AgNO_3_ (an inhibitor of ethylene biological response), and 200 mg/L IAA, were added to H_2_O containing 0.1% (*v*/*v*) Tween 20 were applied as treatments, and ddH_2_O with 0.1% Tween 20 was used as the control. The shoot apices of ‘2013–12’ and ‘9–6’ at the four-true-leaf stage (S4) were sprayed four times (continuously) with these chemical solutions once per day, and each treatment consisted of 15 plants with three biological replicates. After treatment for 1 d, the shoot apices of ‘2013–12’ and ‘9–6’ that had been subjected to the different chemical solutions were selected for transcript level detection. The primer sequences used in qRT-PCR are listed in Table S3. The sex of each flower up to node 20 on the main stem was determined as male, female or bisexual, and the first female flower node was also documented.

### 4.6. Gene expression at Different Stages of Flower Development

Pumpkin flower buds were divided into seven different stages of flower development. Buds with lengths of 2–4 mm were considered to be at stage 1 (S1); those with lengths of 6–8 mm, stage 2 (S2); 10–12 mm, stage 3 (S3); 15–17 mm, stage 4 (S4); 20–25 mm and a yellow stigma, stage 5 (S5); 30–35 mm, stage 6 (S6); and those one day before flowering were considered to be at stage 7 (S7). Male and female flower buds from S1 to S7 were collected from the trimonoecious line ‘2013–12’, and the stamen, ovary, stigma and style were detached from male and female flower buds for qRT-PCR. The morphologies of female and male flower buds at S1–S7 are shown in Figure S3.

## 5. Conclusions

In the present study, we investigated different transcript expression patterns between trimonoecious and subandroecious pumpkins. The DEGs between shoot apices containing male buds and female buds were mainly enriched in the ethylene and auxin synthesis and signal transduction pathways. The numbers of female flowers in ‘2013–12’ changed significantly, while the numbers in ‘9–6’ changed slightly after exogenous hormone treatments. The expression levels of *CmaACS7*, *CmaACO1*, *CmaETR1* and *CmaEIN3* were positively correlated with the number of female flowers in ‘2013–12’, and IAA might affect pumpkin sex expression by inducing *CmaACS7* expression and indirectly affecting ethylene production. The variations in gene expression in ‘9–6’, especially *CmaACS7*, were small in amplitude. *CmaACS7* expression in the ovary, stigma and style in flower development also indicated that *CmaACS7* plays an important role in sex differentiation. Based on the expression of key genes, a possible reason for the different sex expression between pumpkin lines ‘2013–12’ and ‘9–6’ was determined. 

## Figures and Tables

**Table 1:** Summary statistics of RNA sequencing (RNA-seq).

Sample ID	Raw Reads (Mb)	Raw Bases (Gb)	Clean Reads (Mb)	Clean Bases (Gb)	Valid Bases (%)	Q30 (%)	GC (%)
MS1.1	38.21	4.78	37.06	4.63	96.95	94.29	45
MS1.2	43.52	5.44	42.17	5.27	96.86	94.57	45
MS1.3	46.69	5.84	45.39	5.67	97.16	94.60	45
MS10.1	46.31	5.79	44.96	5.62	97.05	94.55	45
MS10.2	43.91	5.49	42.54	5.32	96.84	94.47	45
MS10.3	42.03	5.25	40.70	5.09	96.81	94.28	45
FS1.1	48.34	6.04	46.86	5.86	96.90	94.49	45
FS1.2	45.03	5.63	43.67	5.46	96.95	94.51	45
FS1.3	47.38	5.92	45.86	5.73	96.75	94.46	45
FS10.1	43.06	5.38	41.86	5.23	97.17	94.71	45
FS10.2	38.27	4.78	36.83	4.6	96.22	93.89	44.5
FS10.3	42.65	5.33	41.33	5.16	96.87	94.60	44.5

**Table 2:** Differentially expressed genes related to ethylene synthesis and signal transduction in one-‘2013–12’ one-true-leaf stage sample (FS1) vs. 10-leaf stage sample (FS10) and ‘6–9’ one-true-leaf stage (MS10) vs. FS10.

Gene ID	FS1 VS FS10 log_2_FoldChange	MS10 VS FS10 log_2_FoldChange	GO Term
*CmaCh03G005620*	1.14	1.18	1-aminocyclopropane-1-carboxylate oxidase
*CmaCh02G000660*	2.75	0.26	1-aminocyclopropane-1-carboxylate oxidase
*CmaCh01G009990*	−1.43	−0.16	negative regulation of ethylene-activated signaling pathway, auxin efflux transmembrane transporter activity, auxin homeostasis, auxin polar transport, auxin-activated signaling pathway
*CmaCh01G016830*	−1.25	−0.44	negative regulation of ethylene-activated signaling pathway, regulation of abscisic acid-activated signaling pathway, cytokinin-activated signaling pathway
*CmaCh02G014030*	1.82	1.32	ethylene-activated signaling pathway, cytokinin-activated signaling pathway
*CmaCh04G006970*	2.74	1.42	ethylene-activated signaling pathway
*CmaCh04G002620*	1.87	0.15	ethylene-activated signaling pathway
*CmaCh04G012360*	1.69	0.44	ethylene-activated signaling pathway
*CmaCh04G020120*	2.35	0.07	ethylene-activated signaling pathway
*CmaCh04G008070*	−1.66	−0.36	ethylene-activated signaling pathway
*CmaCh04G015210*	1.81	0.74	ethylene-activated signaling pathway
*CmaCh04G004940*	Inf	Inf	ethylene-activated signaling pathway
*CmaCh05G004890*	1.08	0.3	ethylene-activated signaling pathway, cytokinin-activated signaling pathway
*CmaCh05G001690*	1.40	0.10	ethylene-activated signaling pathway
*CmaCh07G005340*	2.45	0.70	ethylene-activated signaling pathway
*CmaCh09G004610*	1.31	0.42	ethylene-activated signaling pathway
*CmaCh09G005960*	−3.13	−0.82	negative regulation of ethylene-activated signaling pathway, gibberellin biosynthetic process
*CmaCh09G008010*	2.02	1.57	response to jasmonic acid and salicylic acid
*CmaCh10G006590*	1.34	0.04	ethylene-activated signaling pathway
*CmaCh11G006380*	1.38	0.49	ethylene-activated signaling pathway
*CmaCh11G008140*	1.01	0.11	ethylene-activated signaling pathway
*CmaCh11G019380*	1.17	0.42	negative regulation of ethylene-activated signaling pathway
*CmaCh11G005470*	1.09	0.20	ethylene-activated signaling pathway
*CmaCh17G008890*	2.32	0.86	ethylene-activated signaling pathway
*CmaCh19G010510*	1.53	0.12	negative regulation of ethylene-activated signaling pathway

**Table 3:** Effects of different hormone treatments on sex differentiation of ‘2013–12’ and ‘9–6’.

Treatment	Female Flowers Per Plant %		First Female Flower Node		Bisexual Flowers Per Plant %	
	2013–12	9–6	2013–12	9–6	2013–12	9–6
Ethrel	72.5 ± 3.5a	5 ± 1a	5.9 ± 0.4c	13.7 ± 1.5a	3 ± 1.5b	0
IAA	67.5 ± 5.5a	4.5 ± 1.5a	6.9 ± 1.5c	14.6 ± 2.3a	5.5 ± 1b	0
AVG	37 ± 3.5c	2 ± 1a	12.5 ± 0.8a	17.1 ± 1.2a	28.5 ± 7.5a	0
AgNO_3_	33.5 ± 0.5c	2 ± 1.2a	13.7 ± 0.9a	17 ± 1.6a	33.5 ± 4a	0
control	51 ± 5b	4 ± 0.8a	10.1 ± 1.6b	15.4 ± 1.2a	27.5 ± 5a	0

Note: values are the mean (±standard error) of 45 plants. Means followed by a different letter in each column are statistically different by Duncan’s new multiple range test at *p* ≦ 0.05.

## Supplementary Materials

The Supplementary Materials are available online at https://www.mdpi.com/1422-0067/20/13/3185/s1.

## Abbreviations

ACC	1-aminocyclopropane-1-carboxylate
ACO	1-aminocyclopropane-1-carboxylate oxidase
ACS	1-aminocyclopropane-1-carboxylate synthetase
ERF	Ethylene-responsive factor
EIN	Ethylene- insensitive
WEI	Weak ethylene insensitive
CTR	Constitutive triple response
RNA-seq	RNA sequencing
*C. maxima*	Cucurbita maxima
*C. pepo*	Cucurbita pepo
*C. moschata*	Cucurbita moschata
GO	Gene ontology
KEGG	Kyoto Encyclopedia of Genes and Genomes
FPKM	Fragments per kilobase of exon per million fragments mapped
bp	Base pairs
DEGs	Differentially expressed genes
MAPK	Mitogen-activated protein kinase
LS1	One-true-leaf stage
LS10	10-leaf stage
qRT-PCR	Quantitative reverse transcription polymerase chain reaction
PCA	Principal component analysis
AVG	Aminoethoxyvinyl glycine
IAA	Indoleacetic acid
