# Supplementary material for: Morphological, Transcriptomic and Hormonal Characterization of Trimonoecious and Subandroecious Pumpkin (Cucurbita maxima) Suggests Important Roles of Ethylene in Sex Expression
Source: Int J Mol Sci. 2019 Jun 28;20(13):3185. doi: 10.3390/ijms20133185 (PMC6651883; doi:10.3390/ijms20133185)
Supplement: Supplementary file 1 [file ijms-20-03185-s001.pdf]

Table S1. Primers used for qRT-PCR analyses.

Gene ID	Forward primer (5' – 3')	Reverse primer (5' – 3')
CmaActin	CCTCTCAATCCCAAAGCTAACAG	CGGCCTGGATAGCAACATACA
CmaACO1	AGCTTAATGGCGAAGAACGAGC	TCCCAGTCAAGATCGTTGACCTC
CmaACO2	GCGGTGCTGGAAGAGATGA	CGGCAAGTCCTGTGGATTG
CmaACO3	CCTGCAACTCCTCAAAGACG	GATACGCTTCCTTCTTCTCCTC
CmaACO4	GGTGGAGGGAGAGGAAGATAAGGG	TCAAACAGTCGCAATTGGATTCGTA
CmaACO5	TCAACATCGGCGACCAAATC	TAGCCACCAGGATACAGAGC
CmaABA2	TCGCCTTACGCAGTTCCAA	GATTGTCTCCGCTGATGTATCG
CmaABA4	CGCATATACTTGGATGTCCTTGT	AATGGTAGTCGCCTTGGTGAT
CmaAP2-1	AGGATCATGGCGAGCAGTAG	TGTGCCTTGAACTTCCTTGAAT
CmaAP2-2	CTTCATTCTCAGCGATACTATTCAG	CCATCTATGTCTTGTAACACCTCTA
CmaAP2-3	GGCACATTCAACCAGGAACA	TCCACCGTATCGCTTGCTT
CmaEIN3	TCAGTGACAGTAGCGATTACGA	GAATTGTTCTCCATGAGTTCTTCCT
CmaERF3	AGCGGCGGTAACAGTTCTT	CTCTAATCTCGGCGGCGTAT
CmaCRF4	TCGTCGGTCGGTGAAGATG	GTCGTCTAACAAGGTCGGAATC
CmaSHN3	GCAGCACGAGCGTATGATC	GAAATTGAAGGAAGAGGGCAGTT
CmaPYL8	GACTTCTCTGCTTCTTCAACCTT	GCTCCTCACCAACGACCAT
CmaYUC8	GCCGTTGGATTCACAAGGAG	GCATCTTCTCAAGCAAGCAGTT
CmaGH3	AGCATAGACACCGACAAGACA	AACTCTCG CATCCTCCTCTG
CmaACS7	AGCAAAACTCTGATGTTCTTCAAGC	TGGATCAAATCTAGCTCDTCCG
CmaETR1	AAAGGAGAGCTGCCTGAGAGTC	CACGACGCTCTATAAGTTCCGA

Table S2. Summary of the unigene assembly results.

Type	Unigenes
Total number (n)	747168
Number of >=500 bp (n)	385177
Number of >=1000 bp (n)	266548
Total Length (bp)	969146015
Max Length (bp)	20293
Min Length (bp)	201
Average Length (bp)	1297.09
N50 (bp)	2871

Table S3. Clean reads mapped to the reference genome.

Sample	Total mapped reads	Multiple mapped	Uniquely mapped	Reads mapped in proper pairs
MS1.1	30061769 (81.37%)	10910833 (29.53%)	19150936 (51.84%)	24448312 (66.18%)
MS1.2	34329788	12815692	21514096	28006116

	(81.68%)	(30.49%)	(51.19%)	(66.63%)
MS1.3	37025494	13782476	23243018	30387308
	(81.84%)	(30.46%)	(51.37%)	(67.17%)
MS10.1	36774314	13726473	23047841	30231554
	(82.05%)	(30.62%)	(51.42%)	(67.45%)
MS10.2	34978262	12873489	22104773	29086460
	(82.49%)	(30.36%)	(52.13%)	(68.60%)
MS10.3	33589504	11944626	21644878	28121220
	(82.78%)	(29.44%)	(53.34%)	(69.31%)
FS1.1	38489322	14532099	23957223	31730056
	(82.40%)	(31.11%)	(51.29%)	(67.93%)
FS1.2	35718338	13239186	22479152	29201582
	(82.05%)	(30.41%)	(51.64%)	(67.08%)
FS1.3	38612999	14458188	24154811	31970704
	(82.52%)	(30.89%)	(51.62%)	(68.32%)
FS10.1	33934484	12552519	21381965	27433032
	(81.34%)	(30.08%)	(51.25%)	(65.75%)
FS10.2	30250761	10545475	19705286	25182990
	(82.39%)	(28.72%)	(53.67%)	(68.59%)
FS10.3	33529612	12234465	21295147	27251420
	(81.39%)	(29.70%)	(51.69%)	(66.15%)

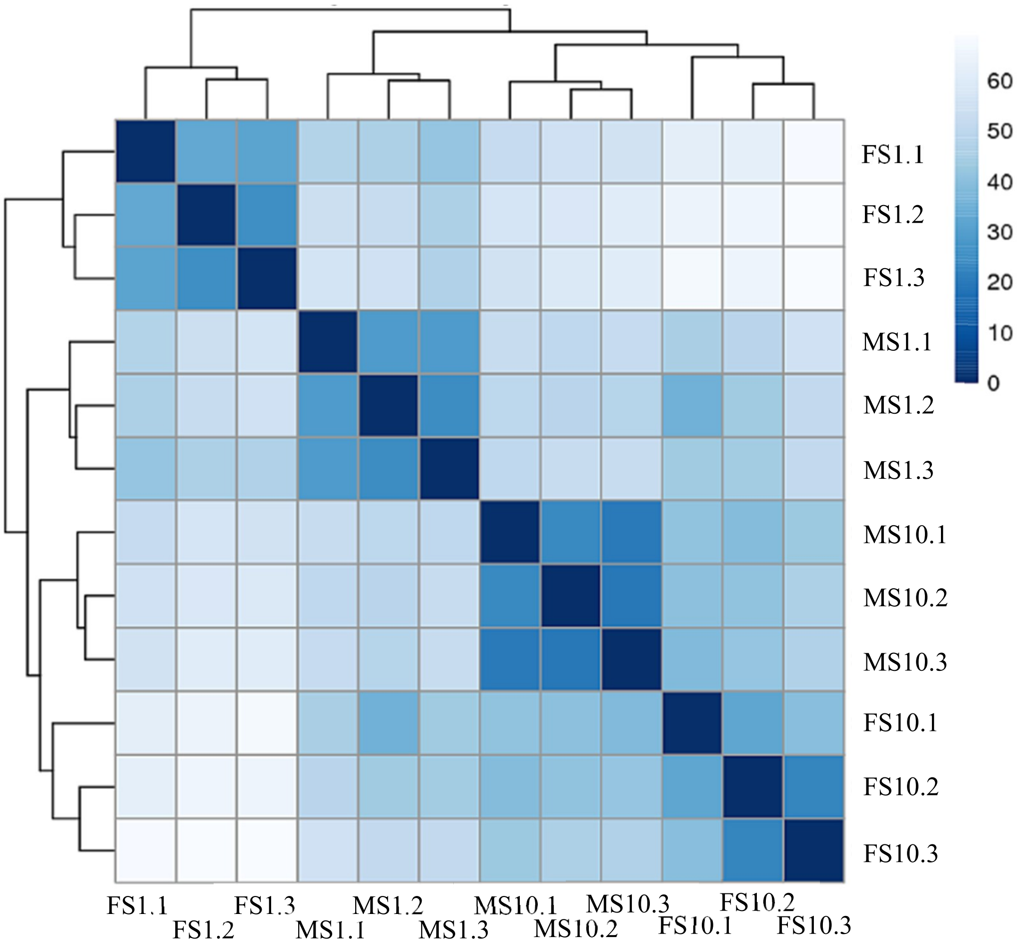

Figure S1. Gene expression distances between replicates on the x-axis and those on the y-axis. The correlation coefficient was show as a color value (white: less similar, and blue: more similar).

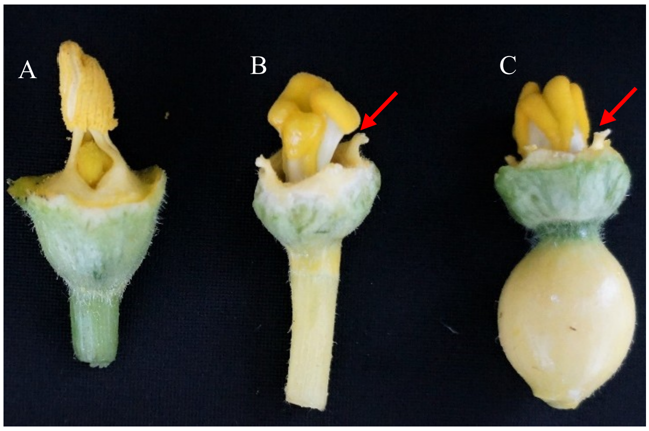

Figure S2. The morphology of normal bisexual flower (A) and female flowers after chemical treatment (B, C). The red arrows indicated underdeveloped stamen of female flowers after chemical treatment.

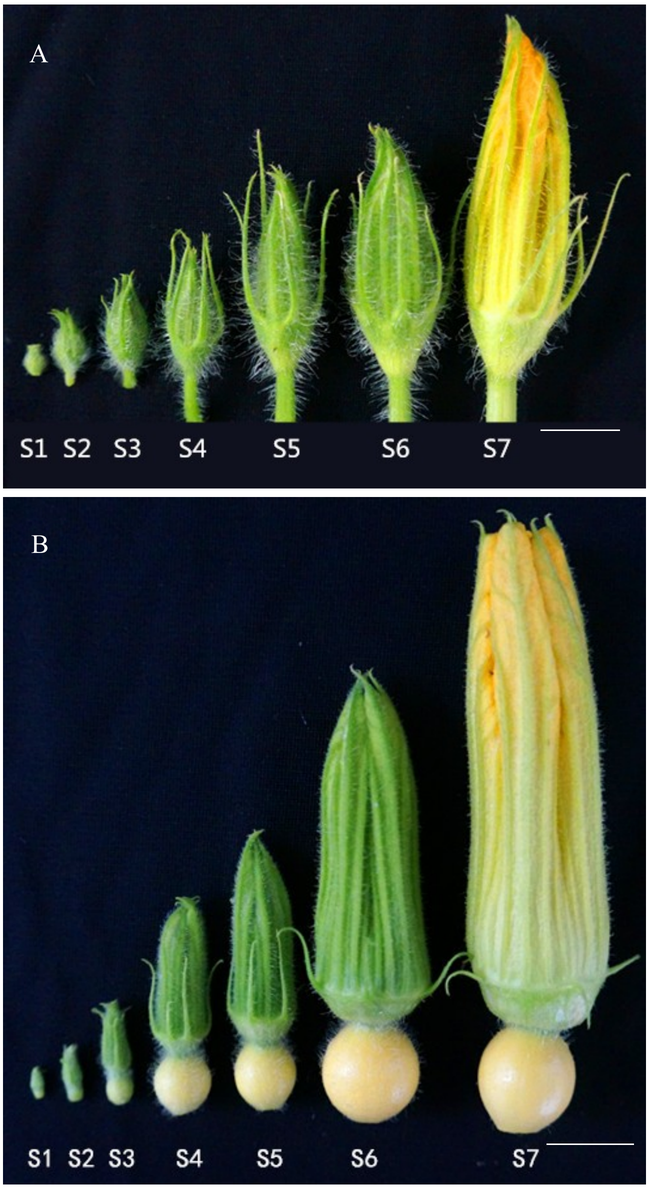

Figure S3. The morphology of female (A) and male flower buds (B) at different stages of flower development. Scale bars in A and B represent 1 cm.
